# Perilla frutescens seed meal as a fat substitute mitigates heterocyclic amine formation and protein oxidation and improves fatty acid profile of pan-fried chicken patties

**DOI:** 10.3389/fnut.2022.975831

**Published:** 2022-09-20

**Authors:** Iftikhar Ali Khan, Baoping Shi, Haibo Shi, Asad Nawaz, Zongshuai Zhu, Muhammad Umair Ijaz, Muzahir Hussain, Asad Khan, Mingfu Wang, Feng Chen, Daoying Wang, Ka-Wing Cheng

**Affiliations:** ^1^College of Civil and Transportation Engineering, Shenzhen University, Shenzhen, China; ^2^Shenzhen Key Laboratory of Marine Microbiome Engineering, Institute for Advanced Study, Shenzhen University, Shenzhen, China; ^3^Institute for Innovative Development of Food Industry, Shenzhen University, Shenzhen, China; ^4^Institute of Agricultural Products Processing, Jiangsu Academy of Agricultural Sciences, Nanjing, China; ^5^School of Food Science and Engineering, South China University of Technology, Guangzhou, China; ^6^Nanjing Innovation Center of Meat Products Processing, Synergetic Innovation Center of Food Safety and Nutrition, College of Food Science and Technology, Nanjing Agricultural University, Nanjing, China; ^7^Department of Medical Pathology and Laboratory Medicine, University of California Davis School of Medicine, Sacramento, CA, United States; ^8^MoBioFood Research Group, Department of Biochemistry and Biotechnology, Universitat Rovira i Virgili, Tarragona, Spain; ^9^Key Laboratory of Mucosal Immunology, College of Preventive Veterinary Medicine, Nanjing Agricultural University, Nanjing, China

**Keywords:** Perilla seed meal, fat substitute, heterocyclic amines, fatty acids, oxidation products

## Abstract

Fatty acid profile, protein and fatty acid oxidation and flavor profile of pan-fried chicken patties formulated with various levels of Perilla frutescens seed meal (PSM) as a fat substitute was investigated in this study. The formation of heterocyclic amines (HCAs) in the chicken patties was also evaluated. The results showed that pan-fried patties formulated with 20% PSM (PSM4) had the highest ranges of oleic acid and ΣMUFA content and ΣPUFA/ΣSFA ratio. Low to medium levels of PSM (PSM1, 2, and 3 corresponding to 5, 10, and 15% of PSM, respectively) reduced the content of lipid peroxidation products, while high level (PSM4) increased it. All levels of PSM were also found to be effective against elevation in carbonyl content relative to the control. Moreover, the PSM effectively inhibited HCA formation in the chicken patties. The total contents of HCAs in PSM1, PSM2, PSM3, and PSM4 samples were significantly (*P* < 0.05) lower than that of the control sample, corresponding to 31.9, 46.1, 57.2, and 44.8% inhibition, respectively. PSM4, however, had no or very little effect on the formation of PhIP, 4,8-DiMeIQx and AαC, despite a strong inhibitory effect on MeIQx formation. These findings not only support the promising potential of PSM for application as a fat substitute to improve the fatty acid profile and reduce the content of harmful by-products in heat-processed chicken, but also highlight that appropriate addition level is a critical factor in optimizing the functional capacity of this natural agent.

## Introduction

Fat is an essential component of the human diet that not only adds flavor to food but also helps to maintain health. However, excessive dietary fat intake has been linked to increased risk of many chronic diseases (1). In particular, a high intake of saturated fatty acids (SFAs) may increase the risk of cardiovascular disease, whereas consumption of sufficient quantities of unsaturated fatty acids (UFAs) may help to reduce it (2). Thus, substituting UFAs for SFAs or adding UFAs has become a popular approach to improving the fatty acid composition of meat products. For example, sunflower oil and Perilla seed addition was reported to significantly increase the percentage of polyunsaturated fatty acids (PUFAs) in fat-modified meat products, while olive oil substantially increased the content of monounsaturated fatty acids (MUFAs) (3, 4). Rodriguez-Carpena et al. (4) replaced 50% of the fat in cooked pork patties with olive, sunflower or avocado oil, and found that olive and avocado oil had the additional advantage of enhancing the aroma of the patties compared with the control. Similarly, Gok et al. (1) reported that replacing 50% of the fat in burger meat with poppy seeds increased the level of UFAs and improved overall acceptability. Choi et al. (5) formulated fat-modified pork batters with rice bran and grape seed oil and discovered that the fat-modified batters had similar texture and flavor to the control. Dominguez et al. (6) replaced 100% of the fat with olive oil in pork pate, which considerably increased the level of MUFAs and tocopherol in the final product without changing the physicochemical characteristics. The use of fish oil, olive oil, and a combination of olive and fish oil to replace 25–75% of the pork fat drastically increased the PUFAs content in Spanish salchichon and frankfurter sausages (7, 8).

Under vigorous thermal conditions, however, seed and vegetable UFAs may undergo decomposition and oxidation, giving rise to a wide range of chemicals. For instance, linoleic acid has been linked to the formation of potentially hazardous substances like ketones, aldehydes and free radicals (9, 10). The reactive oxygen species (ROS) generated by UFAs peroxidation may cause Amadori compounds to degrade and form 1- and 3-deoxysones, which are intermediates in the Maillard reaction for pyridines, pyrazines, and Strecker aldehydes. Potent genotoxic heterocyclic amines (HCAs) can also be formed from these reactions (11, 12). Using UPLC-MS/MS, Li et al. (11) investigated the effect of different vegetable oils on the HCA profile in roasted beef patties. They found that grape seed oil, sunflower oil, and walnut oil significantly reduced PhIP and MeIQ levels, whereas soybean oil, rapeseed oil, and peanut oil increased the levels of MeIQ, MeIQx, 4,8-DiMeIQx, IQx, norharman, and harman. Torreya seed oil and rice germ oil were also reported to effectively inhibit the formation of MeIQ, MeIQx, 4,8-DiMeIQx, IQx, and PhIP. Moreover, Khan et al. (13) reported that marinating with a blend of thyme, sesame seeds and sumac decreased the contents of quinoxalines, pyridines, α-carbolines, quinoxalines, δ-carbolines, and γ-carbolines by ∼30–40%. In a more recent study, Erdoğan Özdestan-Ocak (14) showed that addition of rosehip and pumpkin seed oils to meatballs at 1 and 2% (w/w) reduced the contents of HCAs by 20–83 and 22–77%, respectively.

Perilla (Perillae fructus) seeds have a similar oil content (35–45%) to peanut and sesame seeds, which are popular culinary ingredients and oil seeds in Asia (15). In addition to a high content of PUFAs, especially β-linolenic acid, edible seeds and oils like poppy, sunflower and Perilla frutescens seeds are also high in β–carotenes, vitamin E and phenolic compounds, such as quercetin, rosmarinic acid, febrellin and gallic acid (12, 16). These compounds may help mitigate the above mentioned undesirable reactions during cooking (17). Although the impact of adding Perilla seeds on the physicochemical, nutritional and sensory quality of meat products has recently received some attention (16), their potential to attenuate adverse reactions associated with drastic heat treatment has been severely under-explored. The present study therefore aimed to evaluate the effects of substituting chicken fat with Perilla seed meal (PSM) on the fatty acid profile, protein and lipid oxidation, flavor compounds, and HCA formation in pan-fried reduced-fat chicken patties.

## Materials and methods

### Reagents

Fresh yellow-feathered chicken breast (Xianjia, 24 h post-mortem) was obtained from a local supermarket in Jinagsu, China. Seeds of Perilla frutescens were acquired from Baiyouli, Heihe, China. Authentic HCA standards: norharman, harman, 2-amino-1-methyl-6-phenylimidazo[4,5-b]pyridine (PhIP), 2-amino-9H-pyrido[2,3-b]indole (AαC), 2-amino-3,4,8-trimethyl-3H-imidazo[4,5-f]quinoxaline (4,8-DiMeIQx), 2-amino-3,8-dimethylimidazo[4,5-f]quinoxaline (MeIQx) and 2-amino-3-methylimidazo[4,5-f]quinoline (IQ) (Toronto, Downsview, ON Canada) were dissolved in methanol and stored at 4°C. Acetonitrile, dichloromethane, phosphoric acid and methanol (chromatographic grade) were obtained from Merk KGaA (Darmstadt, Germany). Hydrochloric acid, sodium hydroxide, ammonium acetate and ammonia were purchased from SinoPharm Chem-Reagent Company Limited (Shanghai, China). Distilled water was obtained using a Millipore Direct-Q3uv Ultrapure Water distillation unit.

### Perilla seed meal and chicken patty preparation and cooking

Perilla seeds were roasted, simmered gently, allowed to cool, and smashed using a high speed crusher (BJ-500A, Shanghai Baijie Industry Co., Ltd., China). The ground Perilla seeds were extracted with 5 volumes of n-hexane for 3 h, and the defatted Perilla seed meal (PSM) was obtained. Chicken fat and lean chicken breast meat were cut into small pieces, homogenized separately in an electric grinder, and stored in a refrigerator. For sample uniformity, each batch of chicken meat was thoroughly blended and the ground meat was divided into five parts which were added with different proportions of PSM and ground chicken fat as follows: PSM 0% + fat 20% (PSM0), PSM 5% + fat 15% (PSM1), PSM 10% + fat 10% (PSM2), PSM 15% + fat 5% (PSM3), and PSM 20% + fat 0% (PSM4). For sample uniformity, the patties (50 g each) were formed with the aid of mini metal trays (1.5 cm × 6 cm) as shown in Supplementary Figure 1. Three patties were used for each treatment. The patties were subsequently fried in a preheated non-stick pan on a hot plate (190°C) (C21-WT2118, Media, HEGON, China). The surface of the pan was oiled with sunflower oil to prevent the patties from sticking to the pan. The patties were fried for 4 min on each side (flipped every 2 m). A laser infrared thermometer (Intel instruments, AS852B, China) was used to measure the pan surface temperature (190 ± 4°C), and for the measurement of the patties’ core temperature (72 ± 2°C) a probe thermometer (Center, 309-Datalogger thermometer, Taiwan) was used. All experiments were carried out three times. The fried patties from each treatment were homogenized thoroughly to a uniform sample and kept at −18°C until further analysis.

### Characterization of phenolic compounds in Perilla seed meal

Polyphenols in the PSM were identified in a Waters ultra-performance liquid chromatograph coupled with a quadruple time-of-flight Q-TOF-MS (Xevo G2-S, Milford, MA, United States) using the protocol of our recent study (18). Separation of the PSM phenolic compounds was performed with an Aquity UPLC column (BEH C18, 1.7 m, 100 2.1 mm, Waters, United Kingdom). The mobile phase consisted of 1% formic acid (A) and acetonitrile (B). The injection volume was 2 μL and the flow rate was 0.40 mL/min. Gradient elution was carried out as follows: 0 to 1.5 m, 95% A; 1.5 to 10 min, 95–60% A; 10 to 13.5 min, 60–5% A; 13.5 to 16.5 min, 5% A; 16.5 to 16.8 min, 5–95% A; and 16.8 to 18 min, 95% A. Electrospray ionization (ESI) was performed using an ion source temperature of 120°C, a capillary voltage of 0.5 kV, cone gas and desolvation gas flow rates of 50 and 800 L/h, respectively, and a primary mass scan range of m/z 100–1350. The positive mode lock spray reference ion value was m/z 923.190, and the negative mode value was m/z 921.638.

### Fatty acid composition analysis

An automatic fat extractor was used to extract lipids using the Soxhlet method (SZF-06A, Xinjia Electronics Co., Ltd, Shanghai, China). Fatty acid methyl esters (FAMEs) were generated using methanolic potassium hydroxide solution and n-hexane as described by Satchithanandam et al. (19). The FAMEs were analyzed in a gas chromatograph (QP2010 Plus, Shimadzu, Japan) equipped with an FID detector (FID-14C, Shimadzu, Japan) and a TR-FAME column (60 m × 0.25 mm, 0.25 μm). Supelco’s standard mix was used to identify FAs, and the results were expressed as a percentage of total FAs.

### Analysis of volatile compounds

Solid-phase micro extraction (SPME) was conducted to determine the volatile compound (VCs) in the meat samples using the method of Rasinska et al. (20) with minor modifications. The Supelco device was consisted of a 10-mm fused silica fiber and DVB/CAR/PDMS (50/30 mm). For SPME, each meat sample (5 g) was placed in a vial (40 mL) and incubated at 39°C for 30 min in a water bath. The fiber was inserted into the vial and exposed to the sample headspace for 45 min. Subsequently, the fiber was inserted into the GC injection port for analysis of VCs in a GC-MS/MS instrument (TSQ Quantum XLS, Thermo Fisher Scientific, United States) equipped with an HP-5MS silica capillary column (30 m × 0.25 mm, 0.25 μm, Supelco Co., United States). The fiber was held in the injection port for 3 min at 270°C. The GC temperature program was as follows: initial, 45°C for 2 min, increased to 180°C at a rate of 3°C/min, kept for 2 m, and then raised to 240°C at a rate of 10°C/min, and held for 7 min. The MS parameters were as follows: electron impact mode with a 70 eV ionization energy, a scan mode of m/z 50–550, and an ion source temperature of 230°C. Computer matching with the reference mass spectra of the NIST 14 and Wiley 8.0 MS libraries and linear retention indices (RIs) were used to identify the VCs.

### Analysis of thiobarbituric acid reactive substance assay

Contents of thiobarbituric acid reactive substance (TBARS) were determined using the method of Rodriguez-Carpena et al. (4). A 5 g meat sample was mixed with 25 mL of 7.5% trichloroacetic acid and homogenized for 1 min at 7,000 rpm (T25 digital Ultra-Turrax, IKA, Germany). The supernatant was filtered using Whatman Grade 1 filter paper after centrifugation (10,000 *g*) at 4°C for 10 min. Subsequently, the filtrate was combined with 2 mL of 0.02 M thiobarbituric acid, vortexed, and heated in a water bath for 45 m at 95°C. After cooling, chloroform was added and the mixture was vortexed and then centrifuged. The absorbance of the upper layer was measured at 532 nm. 1,1,3,3-Tetraethoxypropane was used to create a standard curve, and the values were expressed as milligram malondialdehyde (MDA)/kg of meat.

### Carbonyl content analysis

Carbonyl contents of the samples were analyzed using the dinitrophenylhydrazine (DNPH) carbonyl assay (4, 18). A 1 g meat sample was homogenized for 30 s in sodium phosphate buffer with sodium chloride (0.6 M, 20 mM, pH 6.5). The homogenate (2 mL) was split into two equal aliquots and placed in Eppendorf tubes. Following centrifugation at 5,000 rpm for 5 min, 1 mL of 10% trichloroacetic acid was added to each aliquot to precipitate proteins. One of the aliquots was treated with HCl (2 M, 1 mL), while the other with DNPH (1 mL, 0.2%) in HCl (2 M). The samples were incubated at room temperature for 1 h, precipitated again using trichloroacetic acid, and rinsed with 1 mL of ethanol and ethyl acetate (1:1) to eliminate extra DNPH. The precipitate was suspended in sodium phosphate buffer (1.5 mL, 20 mM) containing 6 M guanidine-HCl (pH 6.5), mixed well, and centrifuged at 5,000 rpm for 2 min. BSA (bovine serum albumin) was used as a standard to determine protein concentration at 280 nm. Carbonyl content was expressed as nmol of carbonyl/mg of protein by a protein hydrazone absorption coefficient of 21.0 nM^–1^ cm^–1^ at 370 nm.

### Heterocyclic amine analysis

Heterocyclic amines of the pan-fried chicken patties were extracted using the procedure outlined in our recent investigation (21). The patties were ground and 2 g of each sample was combined with 10 mL of ethylacetate and 2 mL of 1 M NaOH. After homogenization, the sample was centrifuged at 5,000 *g* for 5 min. The supernatant was collected and evaporated under a stream of nitrogen gas. Subsequently, 6 mL of dichloromethane was added and the solution was loaded into a preconditioned PRS cartridge. The PRS cartridge was rinsed sequentially with 6 mL of 0.1 M HCl, 15 mL of methanol/0.1 M HCl (50:50), and 2 mL of distilled water, followed by elution with ammonia solution (0.5 mL) into a preconditioned (5 mL) C-18 cartridge. The same PRS cartridge was eluted again with ammonium acetate (0.5 M, pH 8.5) into another C-18 cartridge. Then, both of the C-18 cartridges were washed with 1 mL of 9:1 methanol/ammonia solution. The eluate was filtered (0.22 um) prior to analysis by HPLC. Detection and quantification of HCAs were performed using an Agilent HPLC (1260, CA) equipped with a fluorescence detector and a diode array detector. A Zorbax Eclipse Plus C18 column (reversed-phase) (Agilent Tech, United States) was employed for the separation of HCAs. A binary mobile phase consisting of 0.05 M ammonium acetate (pH 3.2) (A) and acetonitrile (B) at a flow rate of 1 mL/min was used with a linear gradient elution program starting with 95 A and 5% B, which was modified to 55 A and 45% B in 35 min. The mobile phase was changed back to its original composition for conditioning of the column prior to next injection. Identification of the HCA peaks in the chromatograms was performed by matching against the retention times and UV-absorption spectra of authentic standards and those obtained from spiked samples. For quantification, contents of HCAs in the samples were determined by using external calibration curves constructed with the corresponding authentic HCA standards. For method validation, limit of detection (LOD), limit of quantification (LOQ), linearity and recovery rates were determined and presented in Table 1. The linearity was calculated using regression analysis (R^2^). Recoveries were evaluated by the standard addition method and linear regression equations were used to evaluate the contents of HCAs in the samples.

**TABLE 1:** Retention time, LOD, LOQ, calibration curve, regression coefficient, spiked concentration and recovery rates of different HCAs.

HCAs	ng/g meat sample						
	
	t_*R*_ (min)	LOD (ng/g)	LOQ (ng/g)	Calibration curve equation	Regression coefficient (ng/ml)	Spiked conc. (ng/g)	Recovery rates (%)
IQ	7.43	0.26	0.93	y = 1.1776 × −4.3073	0.999	20–100	62.93 ± 4.10
MeIQx	10.05	0.11	0.39	y = 3.2278 × 2.6592	0.963	20–100	75.06 ± 6.46
4,8-DiMeIQx	11.89	0.15	0.44	y = 1.5215 × −8.6766	0.999	20–100	83.71 ± 7.51
AαC	12.50	0.09	0.25	y = 2.6784 × −2.7095	0.998	2–10	86.92 ± 4.14
PhIP	13.60	0.17	0.69	y = 4.3045 × −1.7426	0.997	2–10	91.56 ± 5.19
Norharman	15.61	0.06	0.27	y = 9.1319 × −1.3957	0.997	2–10	79.05 ± 4.91
Harman	18.84	0.04	0.18	y = 1.9658 × −1.9840	0.999	2–10	80.20 ± 3.58

### Statistical analysis

Triplicate samples of the pan-fried chicken patties were prepared where specified and examined in analyses. ANOVA was employed to compare treatment groups at 0.05 significance level. For multiple comparisons, the Duncan multiple range test was applied using IBM SPSS 26.0.

## Results and discussion

### Characterization of phenolic compounds in Perilla seed meal by UPLC Q-TOF–MS

UPLC–MS detected 57 phenolic compounds in the PSM, including nine flavones, four flavanols, five flavonones, eleven flavonols, nine isoflavonoids, four hydroxybenxoic acids, six anthocyanins, five hydroxycinnamic acids, one dihydroflavonol, three hydroxyphenylacetic acids and two hydroxyphenylpropanoic acids. Their retention times (tR), observed masses (m/z), responses and actual masses are shown in Table 2. The phenolic compounds identified from the PSM are comparable to those of previous studies (22, 23). The main phenolic compounds in our study having high responses were rosmarinic acid, homovanillic acid, caffeic acid, genistein, apigenin, didymin, gallic acid, luteolin, hispidulin, rhamnetin, and kaempferol. Occhiuto et al. (24) investigated the phytochemical composition of hemp seed meal and discovered a high content of flavanones (43%), followed by flavonols (28%), phenolic acids (17%), flavones (12%), flavanols (6%), and isoflavones (3%). Multescu et al. (25) compared the total phenolic and total flavonoid contents of selected by-products of vegetable oil industry and found that rapeseed meals and sunflower meals had much higher contents than black sesame meals, red grape seed meals and golden flax meals. They further showed that sinapic acid derivatives were the most abundant phenolic compounds in the rapeseed meals. Sunflower seeds are high in phenolic compounds with total phenolic contents ranging from 16.28–20.13 mg GAE/g for sunflower kernel and 14.68–18.24 mg GAE/g for sunflower seeds, respectively (26). Caffeic acids and chlorogenic acid accounted for 70% of the phenolic compounds in sunflower flour (27). Natural polyphenols are known for their often strong antioxidant capacity through H-donating, radical scavenging, and/or pro-oxidant chelation (28). Accumulating studies have shown that the antioxidant activity of natural polyphenols plays an important role both in their beneficial effects against chronic diseases such as cancer and their modulating effects on a range of chemical reactions in food (28, 29).

**TABLE 2:** Characterization and classification of phenolic compounds in Perilla seed meal (PSM).

No	Sub class/Compounds	Neutral mass	Observed m/z	t_R_ (min)	Response	Adducts
**I**	**Flavanols**					
1	(+)-Gallocatechin	306.074	307.079	8.34	8448	+H
2	3-Methoxynobiletin	432.142	433.146	14.85	4876	+H
3	4′,4″-O-Dimethylepigallocatechin 3-O-gallate	486.116	487.122	9.64	36740	+H
4	Procyanidin dimer B4	578.142	579.150	3.41	11864	+H
**II**	**Isoflavonoids**					
1	2′,7-Dihydroxy-4′,5′-dimethoxyisoflavone	314.079	315.087	13.94	11695	+H
2	3′-Hydroxygenistein	286.047	287.054	11.84	1470965	+H, −e
3	Dihydrodaidzein 7-O-glucuronide	432.105	433.111	9.24	8417	+H
4	Genistein	270.052	271.058	13.73	123659	+H
5	Melanettin	284.068	285.076	15.65	2734	+H
6	Glycitein 7-O-glucuronide	460.100	461.108	7.56	10689	+H
7	Glycitein 7-O-glucuronide	460.100	461.109	7.83	10577	+H
8	Irisolidone	314.079	315.087	13.95	11694	+H
9	Sativanone	300.099	301.105	12.34	13241	+H
**III**	**Hydroxycinnamic acid**					
1	Caffeoyl tartaric acid	312.048	313.053	2.61	41753	+H
2	Caffeic acid	180.163	179.030	7.07	42575	+H
3	Caffeic acid ethyl ester	208.073	209.078	8.31	53513	+H
4	p-Coumaric acid ethyl ester	192.078	193.084	11.14	5584	+H
5	Rosmarinic acid	360.084	361.090	9.61	153173	+H
**IV**	**Hydroxyphenylacetic acid**					
1	3,4-Dihydroxyphenylacetic acid	168.042	169.047	1.81	13020	+H
2	Dihydro-p-coumaric acid	166.062	167.063	3.57	13612	+H
3	Homovanillic acid	182.057	183.068	5.12	10614	+H
**V**	**Hydroxyphenylpropanoic acid**					
1	3-Hydroxy-4-methoxyphenyllactic acid	212.068	213.075	5.28	7618	+H
2	Dihydroferulic acid 4-sulfate	276.030	277.037	13.22	12725	+H, −e
**VI**	**Hydroxybenzoic acid**					
1	3,4-O-Dimethylgallic acid	198.052	199.058	2.30	19116	+H
2	3-O-Methylgallic acid	184.037	185.041	1.16	10700	+H
3	Syringic acid	198.052	199.056	4.67	28334	+H
4	Gallic acid	170.021	170.021	1.34	23824	−e
**VII**	**Flavanones**					
1	6-Prenylnaringenin	340.131	341.136	7.70	10150	+H
2	Didymin	594.194	595.201	4.58	14609	+H
3	Eriodictyol 7-O-glucoside	450.116	451.123	10.03	6568	+H
4	6-Prenylnaringenin	340.131	341.137	12.81	14120	+H
5	Poncirin	594.194	595.201	4.58	14609	+H
**VIII**	**Flavones**					
1	Apigenin 7-O-diglucuronide	622.117	623.124	7.02	61556	+H
2	Apigenin 7-O-glucuronide	446.084	447.092	9.31	90005	+H
3	Chrysoeriol 7-O-(6″-malonyl-glucoside)	548.116	549.123	11.33	16527	+H
4	Cirsilineol	344.089	345.096	10.28	5503	+H
5	Hispidulin	300.063	301.069	14.33	565379	+H
6	Luteolin 7-O-diglucuronide	638.111	639.119	5.94	14630	+H
7	Luteolin 7-O-glucuronide	462.079	463.086	7.72	31601	+H
8	Luteolin 7-O-glucuronide	462.079	463.086	7.87	97210	+H
9	Pebrellin	344.089	345.095	11.20	9554	+H
**IX**	**Anthocyanins**					
1	Cyanidin 3-O-sophoroside	611.161	611.161	5.81	10640	−e
2	Delphinidin 3-O-(6″-p-coumaroyl-glucoside)	611.140	611.138	8.34	14772	−e
3	Pelargonidin 3-O-sambubioside	565.155	565.153	5.98	6472	−e
4	Pelargonidin 3-O-sophoroside	595.166	595.165	5.40	97442	−e
5	Peonidin 3-O-(6″-acetyl-glucoside)	505.134	505.131	8.34	28952	−e
6	Peonidin 3-O-sophoroside	625.176	625.176	5.61	12471	−e
**X**	**Flavonols**					
1	Isorhamnetin 7-O-rhamnoside	478.111	479.118	9.09	5661	+H
2	3-Methoxynobiletin	432.142	433.147	6.36	7101	+H
3	5,4′-Dihydroxy-3,3′-dimethoxy-6:7-methylenedioxyflavone 4′-O-glucuronide	534.101	535.107	11.20	27327	+H
4	Isorhamnetin 3-O-glucoside 7-O-rhamnoside	624.169	625.179	5.78	6078	+H
5	Kaempferol 3,7-O-diglucoside	610.153	611.161	9.14	5208	+H
6	Kaempferol 3-O-galactoside	448.100	449.106	6.81	224849	+H
7	Myricetin 3-O-arabinoside	450.079	451.089	6.99	6113	+H
8	Patuletin 3-O-glucosyl-(1- > 6)-[apiosyl(1- > 2)]-glucoside	788.201	789.210	12.66	5102	+H
9	Quercetin 3-O-arabinoside	434.084	435.090	8.79	15733	+H
10	Quercetin 3-O-(6″-malonyl-glucoside)	550.095	551.103	1.22	6897	+H
11	Rhamnetin	316.165	317.156	9.65	123785	+H
**XI**	**Dihydroflavonols**					
1	Dihydroquercetin 3-O-rhamnoside	450.116	451.123	10.03	6568	+H

### Perilla seed meal modulates fatty acid profiles of pan-fried chicken patties

The result from the GC analysis showed that the PSM used in the current study had a much higher content of UFAs (90.45%) than SFAs (8.314%) (Table 3), which are in agreement with the data from Perilla frutescens seed oil in Ding and co-workers’ study (30). Incorporation of the PSM to chicken patties significantly influenced the fatty acid profiles of the pan-fried chicken patties relative to the control. In particular, the contents of UFAs in the chicken patties were positively correlated with the levels of PSM added, whereas a negative correlation was found between the addition levels of PSM and SFA contents. Ran et al. (16) found that the use of Perilla seed as a fat replacer could result in increased level of UFAs and decreased level of SFAs in meatballs. In the present study, among the fatty acids determined in the patties, the relative percentage of oleic acid (c18:1n9c) increased from 18.37% in the control (PSM0) group to 35.11% in the PSM4 group, which was likely due to the high content of oleic acid in the PSM, whereas a mild reduction was noted in the content of linolenic acid (c18:2n6c). Gok et al. (1) substituted animal fat in meat burger with poppy seed paste, which resulted in significantly lower contents of SFAs. In all the PSM-treated groups, the proportions of total MUFAs and PUFAs increased significantly (*P* < 0.05) in comparison with SFAs. Of note, UFAs occupied >90% of the total FAs in the PSM, which likely contributed to the dose-dependent increase in the UFA levels in the PSM-added chicken patties. Modern Westernized diets are known to have severely imbalanced contents of FAs with predominantly more SFAs relative to UFAs. Meanwhile, accumulating evidence tends to support a beneficial association between dietary intake of UFAs (especially MUFAs and PUFAs) and the risks of cardiovascular diseases (4, 31). The MUFA and PUFA levels of the PSM4 patties were 27.81 and 37.72%, respectively. The ratio of PUFAs to SFAs increased considerably (*P* < 0.05) as the quantity of PSM increased, which agrees well with the result of Ran et al.’s study (16). According to Liu et al. (32), increasing the ratio of PUFAs/SFAs in beef products could improve lipid and protein oxidative stability. On the other hand, promoting fatty acid oxidation while reducing carbohydrate oxidation as part of the metabolic response to dietary lipid intake (MUFA-rich foods) has been reported to be associated with weight loss (33). In the present study, the significant increase in UFAs content, especially MUFAs, along with the significant decrease in SFAs content of the chicken patties added with the PSM may help to modulate dietary fatty acid intake from the perspective of a balanced dietary lipid intake in favor of reduction in metabolic disease risks (34).

**TABLE 3:** Fatty acid composition (%) of PSM and pan-fried chicken patties incorporated with different levels of Perilla seed meal (PSM).

Fatty acids	PSM	PSM 0%	PSM 5%	PSM 10%	PSM 15%	PSM 20%
c14:0	nd	0.071 ± 0.002^b^	0.082 ± 0.003^b^	0.061 ± 0.005^b^	0.085 ± 0.006^b^	1.220 ± 0.120^a^
c14:1	nd	nd	nd	nd	nd	0.274 ± 0.006
c15:0	nd	nd	nd	nd	nd	0.296 ± 0.005
c15:1	nd	nd	nd	5.911 ± 0.20^a^	5.650 ± 0.19^b^	Nd
c16:0	6.042	8.934 ± 0.311^c^	9.42 ± 0.110^b^	2.932 ± 0.211^e^	3.964 ± 0.20^d^	17.179 ± 0.301^a^
c16:1	0.126	0.428 ± 0.021^b^	0.505 ± 0.031^b^	0.297 ± 0.011^b^	0.521 ± 0.021^b^	2.217 ± 0.303^a^
c17:0	nd	0.054 ± 0.011^b^	0.064 ± 0.005^b^	0.058 ± 0.008b	0.054 ± 0.015^b^	0.667 ± 0.189^a^
c17:1	nd	0.035 ± 0.004^b^	0.044 ± 0.006^b^	0.042 ± 0.010^b^	0.039 ± 0.004^b^	0.452 ± 0.029^a^
c18:0	2.121	2.641 ± 0.289^b^	2.986 ± 0.201^b^	2.659 ± 0.189^b^	3.072 ± 0.10^b^	11.06 ± 0.70^a^
c18:1n9t	nd	0.039 ± 0.009	nd	nd	nd	nd
c18:1n9c	17.894	18.372 ± 0.51^c^	22.272 ± 0.511^b^	18.116 ± 0.302^c^	22.781 ± 0.399^b^	35.114 ± 0.899^a^
c18:2n6c	11.425	28.747 ± 0.501^a^	26.021 ± 0.60^b^	28.892 ± 0.531^a^	26.677 ± 0.55^b^	24.639 ± 0.40^c^
c20:0	0.151	0.168 ± 0.004^c^	0.196 ± 0.016^ab^	0.175 ± 0.010^ab^	0.20 ± 0.008^ab^	0.220 ± 0.050^a^
c18:3n6	0.230	0.327 ± 0.009^a^	0.304 ± 0.008^ab^	0.337 ± 0.014^a^	0.32 ± 0.070^ab^	0.259 ± 0.008^b^
c20:1	0.231	nd	nd	nd	nd	0.391 ± 0.013
c18:3n3	60.543	nd	nd	nd	nd	2.623 ± 0.131
c21:0	nd	38.076 ± 0.992^a^	35.962 ± 0.599^b^	38.647 ± 0.511^a^	34.075 ± 0.41^c^	0.376 ± 0.019^d^
c20:2	nd	0.049 ± 0.004^b^	0.068 ± 0.011^a^	0.054 ± 0.004^b^	0.069 ± 0.005^a^	nd
c22:0	nd	0.066 ± 0.004^d^	0.383 ± 0.011^b^	0.15 ± 0.002c	0.77 ± 0.040^a^	nd
c20:3n6	nd	nd	0.035 ± 0.002^b^	0.032 ± 0.001^c^	0.039 ± 0.001^a^	nd
c20:4n6	nd	0.09 ± 0.010^e^	0.176 ± 0.002^d^	0.192 ± 0.003^c^	0.206 ± 0.004^b^	0.292 ± 0.003^a^
ΣSFA	8.314	50.045	49.137	44.724	42.579	31.744
ΣUFA	90.451	48.052	49.381	53.831	55.943	65.535
ΣMUFA	18.252	18.839	22.777	24.324	28.952	37.722
ΣPUFA	72.199	29.213	26.604	29.507	26.991	27.813

Numbers are the means of three independent measurements. Different letters in the same row indicate significant differences (ANOVA, Duncan test, *P* < 0.05).

### Perilla seed meal modulates the profile of volatile flavor compounds in pan-fried chicken patties

GC analysis identified 31 VCs from the pan-fried chicken patties, including 10 aldehydes, 6 alcohols, 2 ketones, 2 esters, 7 hydrocarbons, 3 carboxylic acids, and 1 hydroquinone (Table 4). The VC compositions of the control and the PSM-added samples were similar. Although not reaching statistical significance, moderately lower contents of VCs were observed in the PSM-added samples compared with the control. Oxidative cleavage of UFAs during heating could give rise to a mixture of aldehydes and alcohols. Due to the antioxidant properties of the phytochemical constituents in the PSM, its addition to the chicken patties would help to suppress ROS generation and scavenge free radicals. The moderately (though not statistically significant) lower contents of hexanal, heptanal octanal, decanal and tridecanedial in the PSM-treated samples (PSM1, PSM2, PSM3, and PSM4) compared with that of the control might partly explain the protective effect of the PSM antioxidant phytochemicals against heat-induced oxidative degradation reactions in the chicken patties. Hexanal, in particular, is an essential criterion for assessing meat flavor. Low hexanal levels are associated with bready and fruity flavors, while high levels are associated with rancid and fatty flavors (35, 36). Ran et al. (16) also found that adding Perilla seeds as a partial fat substitute reduced hexanal content in meatballs from 16.30 to 0.49%. In contrast, Sabikun et al. (37) found that as milk fat and potato mash levels were increased, aroma compounds such as nonanal, hexanal, 2-hexanol, 2-heptanone, 2-pentanone, 1-octen-3-ol, and 3-methylbutanal were elevated in chicken nuggets. Similarly, in a more recent study, it was reported that the addition of 2% white ginseng could increase the variety and relative contents of aldehydes, hydrocarbons, ketones, and esters in roasted chicken (38). Moreover, the finding from Carvalho et al.’s study (39) that pitanga and guarana extract-added lamb burger exhibited slightly lower levels of VCs was in agreement with our results. These data together indicate that incorporating PSM into meat patties may be an effective approach to preventing the generation of certain lipid oxidation products that are known to have a negative impact on meat flavor. Furthermore, PSM may offer an additional benefit by functioning as a source of antioxidants and dietary fiber (16, 40).

**TABLE 4:** Relative content^a^ of volatile compounds in pan-fried chicken patties incorporated with different levels Perilla seed meal (PSM).

S. No	Volatile compounds	Formula	t_R_	PSM 0%	PSM 5%	PSM 10%	PSM 15%	PSM 20%
**I**	**Aldehydes**							
1	Hexanal	C_6_H_12_O	4.28	0.200	0.182	0.133	0.124	0.199
2	Heptanal	C_7_H_14_O	6.41	0.012	0.007	0.008	0.004	0.002
3	Benzaldehyde	C_7_H_6_O	7.86	0.051	0.023	0.012	0.012	0.051
4	Octanal	C_8_H_16_O	8.58	0.141	0.126	0.116	0.106	0.022
5	Benzeneacetaldehyde	C8H8O	8.76	0.009	0.011	0.024	0.017	0.009
6	Non-anal	C_9_H_18_O	9.60	0.080	0.095	0.070	0.069	0.080
7	Decanal	C_10_H_20_O	12.99	0.013	0.008	0.008	0.010	0.013
8	Undecanal	C_11_H_22_O	13.87	0.004	0.003	0.006	0.004	0.005
9	Dodecanal	C_12_H_24_O	17.57	0.024	0.038	0.093	0.129	0.024
10	Tridecanedial	C_13_H_24_O_2_	25.92	0.009	0.006	0.005	0.003	0.002
	**Total aldehydes**			**0.543**	**0.499**	**0.475**	**0.478**	**0.407**
**II**	**Alcohols**							
1	1-Hexanol	C_8_H_18_O	18.06	0.037	0	0	0	0
2	1-Heptacosanol	C_27_H_56_O	18.45	0.005	0.002	0.001	0.001	0.001
3	13-Heptadecyn-1-ol	C_17_H_32_O	24.14	0.005	0.007	0.006	0.004	0.006
4	Cyclopentanol	C_15_H_22_O	13.70	0.002	0.003	0.003	0.002	0.003
5	1-Hexadecanol	C_17_H_36_O	17.76	0.005	0.005	0.008	0.009	0.009
6	Cyclohexanol	C_10_H_20_O	8.41	0.011	0.015	0.016	0.019	0.021
	**Total alcohols**			**0.065**	**0.032**	**0.034**	**0.035**	**0.04**
**III**	**ketones**							
1	2-Hexanoylfuran	C_10_H_14_O_2_	18.22	0.009	0.002	0.001	0.006	0.009
2	2,3-Octanedione	C_8_H_14_O_2_	19.27	0.038	0	0	0	0
	**Total ketones**			**0.047**	**0.002**	**0.001**	**0.006**	**0.009**
**IV**	**Esters**							
1	Butyl benzoate	C_11_H_14_O_2_	19.53	0.012	0	0	0	0
2	7-Methyl-Z-tetradecen	C_17_H_32_O_2_	14.96	0.002	0.003	0.004	0.003	0.002
	**Total easters**			**0.014**	**0.003**	**0.004**	**0.003**	**0.002**
**V**	**Hydrocarbons**							
1	4,4-Dipropylheptane	C_13_H_28_	23.18	0.005	0.003	0.002	0.005	0.002
2	Hexadecane	C_16_H_34_	24.46	0.025	0.004	0.004	0.025	0.003
3	Undecimal	C_12_H_26_	9.93	0.001	0.070	0.066	0.064	0.063
4	Dodecane	C_12_H_26_	11.40	0.012	0.011	0.010	0.010	0.011
5	Tetradecane	C_15_H_32_	15.56	0.006	0.013	0.011	0.009	0.013
6	Hexadecane	C_16_H_34_	16.22	0.025	0.003	0.002	0.002	0.003
7	Tetradecane	C_14_H_30_	16.82	0.085	0.044	0.042	0.032	0.023
	**Total hydrocarbons**			**0.159**	**0.148**	**0.137**	**0.147**	**0.118**
**VI**	**Carboxylic acids**							
1	Decanoic acid	C_20_H_40_O_2_	20.56	0.104	0.100	0.102	0.102	0.107
2	Dodecanoic acid	C_12_H_24_O_3_	22.27	0.003	0.008	0.009	0.008	0.005
3	n-Hexadecanoic acid	C_16_H_32_O_2_	22.57	0.050	0.026	0.027	0.026	0.024
	**Total carboxylic acids**			**0.157**	**0.134**	**0.138**	**0.136**	**0.136**
**VII**	**Hydroquinones**							
1	1,4-Benzenediol	C_14_H_22_O_2_	28.76	0.015	0.002	0.001	0.001	0.001

^a^The values represent the contents of the volatile compounds relative to the content of the total volatile compounds in the control.

### Perilla seed meal modulates lipid oxidation in pan-fried chicken patties

**FIGURE 1:**
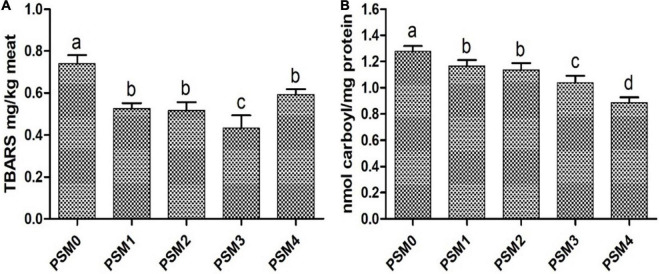
TBARS **(A)** and carbonyl **(B)** content of pan-fried chicken patties formulated with various levels of Perilla seed meal (PSM) as a fat substitute (*P* < 0.05). PSM 0% + fat 20% (PSM0), PSM 5% + fat 15% (PSM1), PSM 10% + fat 10% (PSM2), PSM 15% + fat 5% (PSM3), and PSM 20% + fat 0% (PSM4). Different letters above the bars indicate significant difference at *P* < 0.05.

As presented in Figure 1A, the TBARS values of all the PSM-added patties were significantly (*P* < 0.05) lower than that of the control. The phenolic compounds found in the PSM could play a major role in the inhibition of lipid oxidation. Wong et al. (41) reported that certain vitamins, in particular vitamins A, B6, C and E, could effectively inhibit lipid peroxidation in pan-fried beef patties as supported by the significantly reduced contents of a major lipid peroxidation product MDA compared to control. Apart from widely known action mechanisms such as scavenging of peroxyl radicals or singlet oxygen, direct trapping/adduction reaction with reactive intermediates or products such as MDA has also been suggested to contribute significantly to attenuation of the content of hazardous lipid peroxidation products (42). Polyphenols such as procyanidins and epicatechin have also been found to be effective in blocking chain propagation and inhibiting lipid oxidation in pork and beef (42, 43). Nain et al. (44) found that green tea extract was more effective than α-tocopherol in preventing oxidative deterioration of DHA-rich oil. In our study, the high-dose PSM (PSM4) was less effective in inhibiting lipid oxidation (TBARS) than the medium dose (PSM3) in pan-fried chicken patties. This was likely due to the higher content of UFAs in PSM4 than in PSM3, and the susceptibility of the UFAs to peroxidation might outweigh the potential inhibitory effect of the purported antioxidant phytochemicals.

### Perilla seed meal modulates protein oxidation in pan-fried chicken patties

Protein carbonyl content, a widely used marker of protein oxidation, was measured to assess the levels of protein oxidation in the pan-fried chicken patties (Figure 1B). All the four levels of the PSM could significantly (*P* < 0.05) reduced the content of protein carbonyls in the chicken patties compared with the control. Moreover, the inhibitory effect exhibited a dose-dependent trend with PSM4 showing the highest inhibition rate at 30.49% relative to the control. The protective effect of the PSM against protein oxidation in the chicken patties may be partly ascribed to its phenolic constituents. Botsoglou et al. (45) reported that adding 50 mg/kg α-tocopherol to pork patties significantly reduced protein carbonyl levels. Similarly, Ganhao, Morcuende, and Estevez (46) found that chelating heme iron with catechin-rich arbutus berry extract greatly decreased protein oxidation in burger patties. It was also shown that sinapic acid and vinylsyringol in pitanga leaf extract and cyaniding and luteolin in rapeseed oil could suppress the formation of oxidation products in pork patties and burger, respectively (47, 48). Khan et al. (22) suggested that phenolic compounds found in chrysanthemum morifolium flower extract, such as rosmarinic acid, caffeic acid and quercetin, could protect against protein denaturation in goat meat patties. Heat-induced alteration in protein conformation could lead to the exposure of more amino acid side chains, whose oxidation in meat products has been suggested to be a primary cause of protein carbonylation (18). Carbonylation is a non-enzymatic process that can result in irreversible changes in protein structure, and examples of amino acids susceptible to the above mentioned direct oxidation reaction and thus carbonylation include proline, arginine, lysine, and histidine (49).

### Perilla seed meal modulates heterocyclic amine formation in pan-fried chicken patties

Polar HCAs, such as MeIQ, MeIQx and PhIP, have long been reported to be among the major contributors to HCA-associated genotoxic/carcinogenic activity in foods. In recent years, non-polar HCAs such as harman, norharman and AαC have also attracted increasing attention considering the limited knowledge available on the full spectrum of the potential health impact of this category of structurally close heat-induced hazardous compounds. In the present study, representative candidates from these two categories based on their relative abundance and frequency of occurrence in foods were selected to investigate the potential of different addition levels of PSM to attenuate HCA content in pan-fried chicken patties. The results are presented in Table 5. The level of IQ was undetectable in all the samples. The levels of the HCAs in the chicken patties were similar to those reported in previous studies. For example, Khan et al. (50) found that 7,8-DiMeIQx, IQ and MeIQx concentrations in cooked meat were lower than the LODs. Puangsombat et al. (51) detected 3.1 ng/g of MeIQx and 2.35 ng/g of PhIP in fried beef patties (204°C, 5 min). In general, the levels of individual HCAs were significantly (*P* < 0.05) lower in the PSM-treated samples compared to the control. The inhibitory effect was particularly strong with regard to the formation of non-polar HCAs harman and norharman. It was noticed that the two medium levels (PSM2 and PSM3) were more effective than the low (PSM1) and the high (PSM4) levels against the formation of PhIP, 4,8-DiMeIQx and AαC. An exception was MeIQx, whose formation was most effectively suppressed by PSM4. This pattern was similar to that observed in the lipid oxidation assay (Section 3.4). Further study would be needed to investigate the potential connection (if any) between the PSM-modulated lipid oxidation and HCA formation pathways in the chicken patties. The total HCA contents of the PSM1, PSM2, PSM3, and PSM4 samples were 32, 46, 57, and 45% lower than that of the control, respectively. The stronger effect of PSM3 than PSM4 against the formation of HCAs, especially PhIP and AαC reinforced the notion that appropriate addition level is a critical factor in optimizing the inhibitory efficiency of natural agents. Hsu and Chen (31) examined the levels of HCAs in chicken skin and meat, and found higher levels in the former than the latter tissue. They further showed that rosemary was more effective than black pepper, red pepper and soy sauce against HCA formation. Cheng et al. (52) investigated the effect of sugarcane molasses extract on HCA formation in deep-fried chicken wings, and observed ∼18.5–43.9% reduction in the HCA levels in the extract-added samples compared to the control. Using strawberry, raspberry, and blueberry marinades, Khan et al.’s study achieved 40–67% inhibition of the formation of β-carbolines and 91–100% inhibition of the formation of pyridines, quinolines, α-carbolines, γ-carbolines, and quinoxalines in chicken, beef, and camel meat (53).

**TABLE 5:** Contents of different HCAs in pan-fried chicken patties incorporated with various levels of Perilla seed meal (PSM).

HCAs	ng/g meat sample				
	
	PSM 0%	PSM 5%	PSM 10%	PSM 15%	PSM 20%
IQ	nd	nd	nd	nd	nd
MeIQx	2.47 ± 0.47^a^	1.63 ± 0.43^bc^	2.33 ± 0.55^ab^	1.24 ± 0.15^c^	nd
4,8-DiMeIQx	3.12 ± 0.25^a^	3.05 ± 0.58^a^	2.73 ± 1.05^ab^	1.78 ± 0.32^b^	2.75 ± 0.37^ab^
Norharman	3.23 ± 0.93^a^	1.80 ± 0.25^b^	0.72 ± 0.14^c^	0.97 ± 0.21^c^	0.89 ± 0.05^c^
Harman	2.85 ± 0.52^a^	0.69 ± 0.15^b^	1.11 ± 0.66^b^	0.59 ± 0.13^b^	0.92 ± 0.51^b^
PhIP	3.47 ± 0.26^a^	3.01 ± 0.61^ab^	1.84 ± 0.48^c^	2.20 ± 0.74^bc^	3.37 ± 0.11^a^
AαC	1.46 ± 0.37^a^	1.12 ± 0.16^a^	0.21 ± 0.01^b^	0.32 ± 0.17^b^	1.23 ± 0.02^a^
ΣHCAs	16.59	11.30	8.94	7.10	9.16
% reduction	−	31.89	46.11	57.20	44.78

Numbers are the means of three independent measurements. Different letters in the same row indicate significant differences (ANOVA, Duncan test, *P* < 0.05).

Phenolic compounds have been among the most important classes of phytochemicals with promising potential for mitigating dietary HCA exposure. Major inhibitory mechanisms identified thus far include the scavenging or trapping of carbon-centered and pyrazine cation radicals generated during the Maillard reaction, and trapping of key reactive intermediary compounds, especially carbonyl species such as phenylacetaldehyde in PhIP formation (10, 54). In the present study, the highest level of PSM did not prove to have any significant advantage over the low addition levels. Besides polyphenols, PSM also contains a variety of other compounds, such as UFAs and polysaccharides (55). Under the vigorous thermal condition, the physicochemical properties and thus interactions among the added phytochemicals and the constituents of the chicken patties might exhibit complex behaviors, which could be modulated in response to multiple factors, such as concentration of the reacting partners, moisture level, and mobility of molecules. This is probably one of the major reasons for the often lack of consistent concentration-response/inhibitory activity relationships in studies involving plant extracts (e.g., seed meals) that have complex phytochemical profiles. Further studies are required to better understand the individual as well as the potentially inter-winding roles of the purported HCA inhibitory constituents in PSM.

### Principle component analysis

**FIGURE 2:**
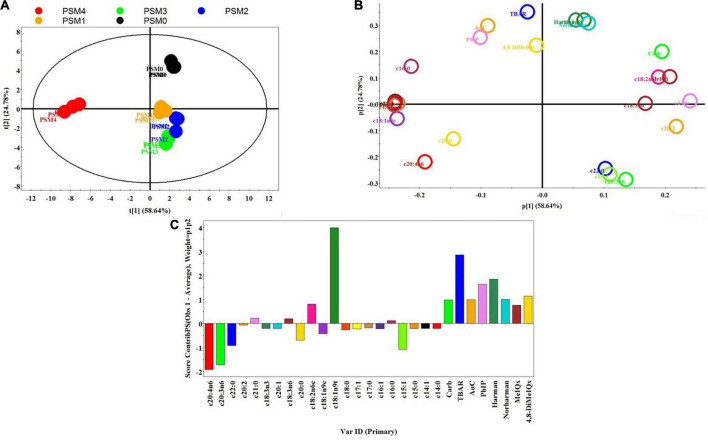
Principal component analysis; **(A)**, score plot (PC1 versus PC2) **(B)**, loading plot **(C)**, contribution plot of HCAs, TBARS, carbonyl and fatty acid profiles of pan-fried chicken patties incorporated with different levels of Perilla seed meal (PSM) as a fat substitute. PSM 0% + fat 20% (PSM0), PSM 5% + fat 15% (PSM1), PSM 10% + fat 10% (PSM2), PSM 15% + fat 5% (PSM3), and PSM 20% + fat 0% (PSM4).

Principle component analysis (PCA) was applied to have a more comprehensive evaluation of the key parameters of the roasted chicken patties that were affected by pretreatment with various levels of PSM, including HCA contents, lipid and protein oxidation, and fatty acid contents. Figures 2A–C show the three plots (score, loading and contribution) for the PSM-pretreated and control samples. The total variance of PC1 and PC2 of the model (83.42%) indicates that the PCA analysis was appropriate for the correlation among the various analyses and that the analysis method was effective. The data points for the control samples are positioned in the upper-right quadrant of the ellipse, whereas those for the low to medium levels of PSM-added samples are located in the lower-right quadrant, indicating that these addition levels of the PSM generated a significant effect on FA, TBARS, carbonyl and HCA contents (Figure 2A). The data points of the PSM4 samples are located opposite and faraway from the control points suggesting that the high level of PSM had the strongest effect on the above parameters. The big distances between the control and the PSM-added samples indicate their highly differentiated profiles of the quality attributes. On the other hand, the small distances among the data points of harman, norharman, c18:1n9t, TBARS, PhIP, AαC and 4,8-DiMeIQx (Figure 2B) in the area where the control points are located in the score plot indicate a high correlation among these parameters. Toward the bottom of the loading plot, the correlation among the different analyses weakens, which could be supported by the heights of the bars (upward or downward direction) in the contribution plot (Figure 2C). As the bar height increased in upward direction, the correlation among parameters increased and as the bar height decreased the correlation decreased, whereas the downward bars indicate negative correlations. For instance, the direction of the c20:4n6, c20:3n6, c15:1, c22:0, and c20:0 bars are downward indicating a negative correlation of these fatty acids with the rest of the parameters analyzed. During cooking or storage, proteins and fatty acids in meat may undergo chemical changes such as hydrolysis and oxidation, which may provide precursors or intermediary compounds to promote HCA formation (56). Herein, the PCA analysis supports a negative correlation between HCA contents and the parameters associated with protein (i.e., carbonyls) and fatty acid oxidation (i.e., TBARS). In a study by Wu et al. (57), the content of harman was also found to be positively correlated with TBARS and carbonyl content, while that of norharman was positively correlated with TBARS. The correlation analysis therefore suggests that protection against protein and fatty acid oxidation likely plays an important role in the inhibitory effect of PSM against HCA formation in roasted chicken patties.

## Conclusion

In conclusion, incorporation of the PSM as a fat substitute could significantly and favorably modulate the profile of fatty acids in pan-fried chicken patties, and the effects of the low and medium levels of the PSM were better than that of the high level of the PSM. Protein oxidation was also significantly attenuated in the PSM-added samples relative to the control. Furthermore, the various levels of the PSM were also able to effectively reduce the content of total HCAs by 31.89–57.20% relative to the control. In terms of inhibition of the formation of individual HCAs, the effects of the two medium levels were generally better than the low and high levels of the PSM, especially with regard to the formation of critical HCAs such as PhIP. The data from the present study not only support the promising potential of PSM for application as a fat substitute to improve the fatty acid profile and reduce the content of harmful by-products in heat-processed chicken, but also highlight that appropriate addition level is a critical factor in optimizing the functional capacity of seed meals. Considering the rich and diverse types of phenolic constituents in PSM, it would be interesting to further identify the principal candidates responsible for the favorable modulating effects, to explore potential synergistic partners, and to characterize the mechanisms which underlie the concentration-dependent phenomenon observed herein.

## Data availability statement

The raw data supporting the conclusions of this article will be made available by the authors, without undue reservation.

## Author contributions

IK, BS, and HS contributed to methodology, investigation, visualization, and writing—original draft. HS, AN, ZZ, MI, MH, and MW contributed to writing—review and editing. FC contributed to resources. DW and K-WC contributed to project administration, supervision, and writing—review and editing. All authors contributed to the article and approved the submitted version.
