# Supplementary figures and images for: Perilla frutescens seed meal as a fat substitute mitigates heterocyclic amine formation and protein oxidation and improves fatty acid profile of pan-fried chicken patties
Source: Front Nutr. 2022 Sep 20;9:975831. doi: 10.3389/fnut.2022.975831 (PMC9530322; doi:10.3389/fnut.2022.975831)

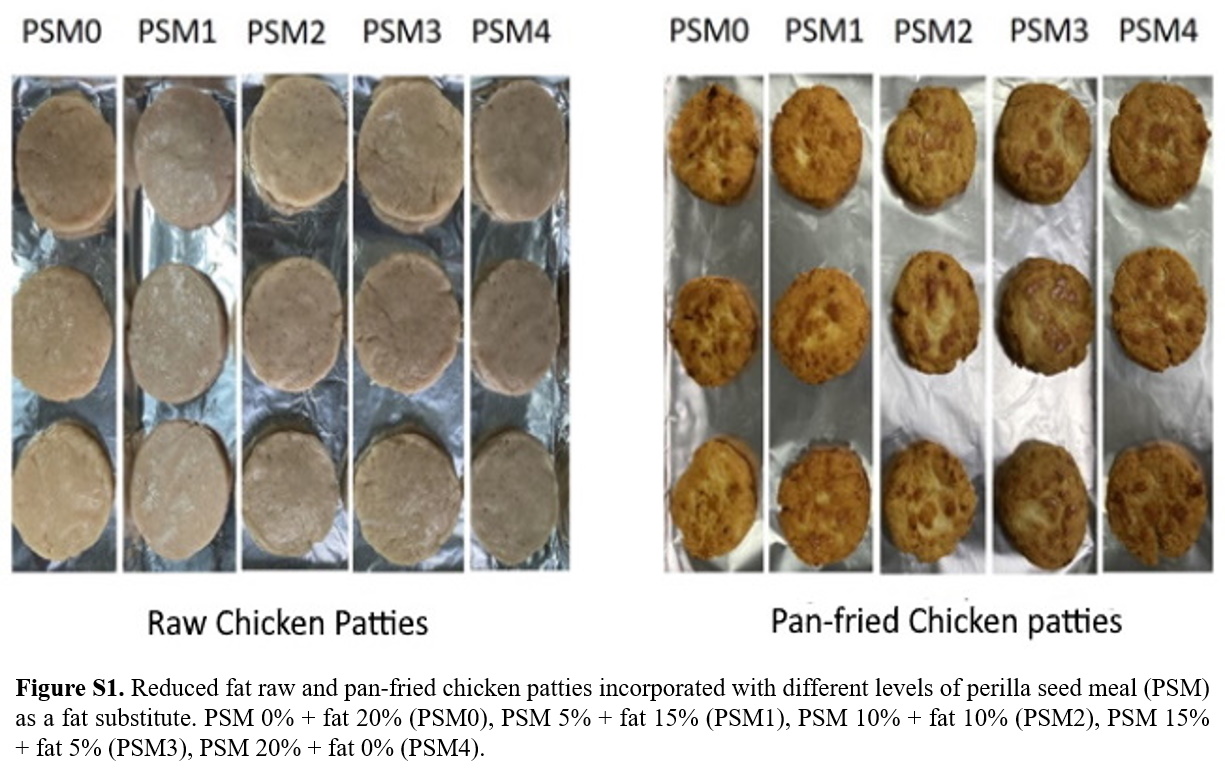

Supplement: Supplementary file 1 [file Image_1.JPEG]
